# Comprehensive renoprotective effects of ipragliflozin on early diabetic nephropathy in mice

**DOI:** 10.1038/s41598-018-22229-5

**Published:** 2018-03-05

**Authors:** Michitsugu Kamezaki, Tetsuro Kusaba, Kazumi Komaki, Yohei Fushimura, Noriko Watanabe, Kisho Ikeda, Takashi Kitani, Noriyuki Yamashita, Masahiro Uehara, Yuhei Kirita, Yayoi Shiotsu, Ryosuke Sakai, Takuya Fukuda, Masahiro Yamazaki, Michiaki Fukui, Satoaki Matoba, Keiichi Tamagaki

**Affiliations:** 1 Department of Nephrology, Graduate School of Medical Science, Kyoto Prefectural University of Medicine, Kyoto, Japan; 2 Department of Cardiovascular medicine, Graduate School of Medical Science, Kyoto Prefectural University of Medicine, Kyoto, Japan; 3 Department of Endocrinology and Metabolism, Graduate School of Medical Science, Kyoto Prefectural University of Medicine, Kyoto, Japan

## Abstract

Clinical and experimental studies have shown that sodium glucose co-transporter 2 inhibitors (SGLT2i) contribute to the prevention of diabetic kidney disease progression. In order to clarify its pharmacological effects on the molecular mechanisms underlying the development of diabetic kidney disease, we administered different doses of the SGLT2i, ipragliflozin, to type 2 diabetic mice. A high-dose ipragliflozin treatment for 8 weeks lowered blood glucose levels and reduced urinary albumin excretion. High- and low-dose ipragliflozin both inhibited renal and glomerular hypertrophy, and reduced NADPH oxidase 4 expression and subsequent oxidative stress. Analysis of glomerular phenotypes using glomeruli isolation demonstrated that ipragliflozin preserved podocyte integrity and reduced oxidative stress. Regarding renal tissue hypoxia, a short-term ipragliflozin treatment improved oxygen tension in the kidney cortex, in which SGLT2 is predominantly expressed. We then administered ipragliflozin to type 1 diabetic mice and found that high- and low-dose ipragliflozin both reduced urinary albumin excretion. In conclusion, we confirmed dose-dependent differences in the effects of ipragliflozin on early diabetic nephropathy *in vivo*. Even low-dose ipragliflozin reduced renal cortical hypoxia and abnormal hemodynamics in early diabetic nephropathy. In addition to these effects, high-dose ipragliflozin exerted renoprotective effects by reducing oxidative stress in tubular epithelia and glomerular podocytes.

## Introduction

Sodium glucose co-transporter 2 inhibitors (SGLT2i) have recently been introduced into clinical practice. Several clinical and experimental studies have shown that SGLT2i exert favorable effects by preventing not only diabetic kidney disease progression, but also cardiovascular disease^1–7^. These studies proposed several underlying mechanisms such as glucose-lowering and pleiotropic mechanisms^1–4^. However, the molecular mechanisms of action of SGLT2i have not yet been elucidated in detail.

Filtered urinary glucose is mainly reabsorbed through SGLT2, which is localized in the apical membrane of the former segment of the proximal tubules. Under diabetic conditions, filtered urinary glucose increases, resulting in the compensatory up-regulation of SGLT2 in tubular epithelia^8,9^. This up-regulation increases glucose and sodium reabsorption, eventually elevating blood glucose levels further and increasing the amount of body fluid. In addition, increased sodium reabsorption by the proximal tubules decreases sodium delivery to the macula densa, which induces glomerular hyperfiltration through the tubulo-glomerular feedback mechanism^10,11^. The excessive entry of glucose into tubular epithelial cells through SGLT has been shown to induce the expression of inflammatory and fibrotic markers^12^. This vicious cycle between the up-regulation of SGLT2 and increases in blood glucose levels and the amount of body fluid synergistically exacerbate the pathophysiology of diabetic nephropathy and may contribute to the progression of kidney dysfunction.

Regarding albuminuria as an initial predictor of early diabetic nephropathy, recent studies showed that the filtration of albumin through the glomerular basement membrane under physiological conditions was more extensive than previously considered, and also that the bulk of albumin was reabsorbed by proximal tubular epithelial cells^13,14^. Therefore, therapeutic strategies that include reductions in glomerular hyperpermeability and the cytoprotection of proximal tubular epithelia and podocytes are needed in order to ameliorate albuminuria in diabetic nephropathy.

In the present study, to comprehensively investigate the renoprotective effects of ipragliflozin, particularly the mechanisms responsible for its glucose-lowering effects, we administered different doses of ipragliflozin to type 2 diabetic model *db/db* mice. Since excessive oxidative stress plays a crucial role in the development of diabetic nephropathy^15^; we analyzed this pathway in tubular epithelia and glomerular podocytes. Previous experimental findings showed that tissue oxygen tension is reduced in diabetic kidneys^16^, and that phlorizin, a non-selective SGLT inhibitor, improves parenchymal oxygenation by reducing sodium reabsorption through SGLTs^17^. Thus, we also focused on the effects of ipragliflozin on oxidative stress and tissue hypoxia in diabetic kidneys.

## Results

### High-dose ipragliflozin improves blood glucose in *db/db* mice

**Figure 1:**
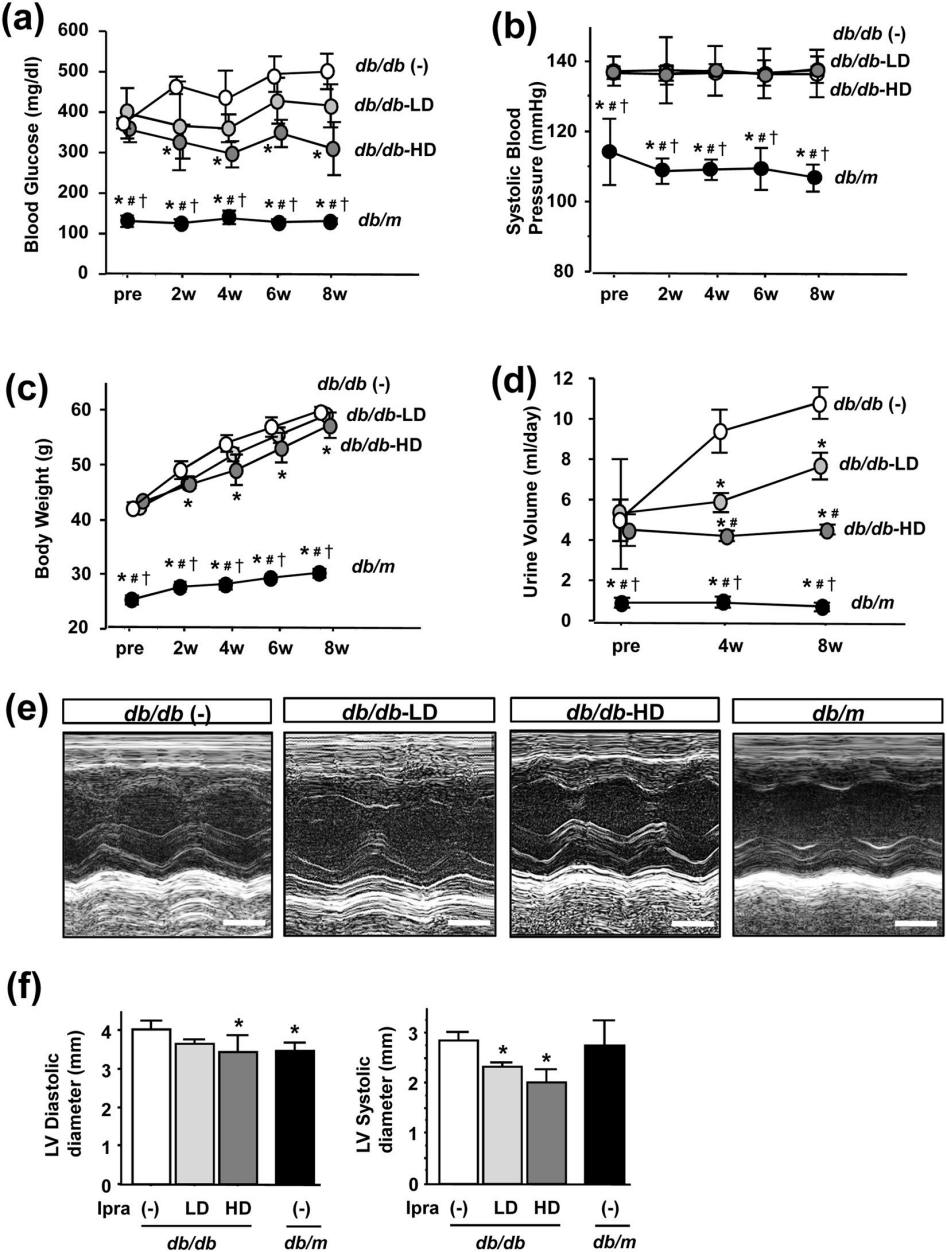
Effects of ipragliflozin on blood glucose levels, body weights, BP, and urine volumes in *db/db* mice. (**a**) High-dose ipragliflozin significantly inhibited further increases in blood glucose levels in *db/db* mice. (**b**) The ipragliflozin treatment did not affect systolic BP. (**c**) High-dose ipragliflozin inhibited body weight gain in *db/db* mice. (**d**) Urine volume was significantly lower in ipragliflozin-treated *db/db* mice. (**e**) Pictures of M-mode echocardiography. (**f**) The ipragliflozin treatment reduced the left ventricular chamber size of *db/db* mice. Data show the means ± SD, *p < 0.05 vs *db/db* (−), ^#^p < 0.05 vs *db/db*-LD, ^†^p < 0.05 vs *db/db*-HD, Bar = 1 mm in (**e**), n = 4–5 mice in each group, one-way ANOVA, Tukey’s multiple comparison.

Four mouse groups were included in this experiment: *db/m* mice as non-diabetic controls, and *db/db* mice treated with a high or low dose of ipragliflozin (3 mg/kg/day: *db/db*-HD, 0.3 mg/kg/day: *db/db*-LD, respectively) or vehicle (*db/db* (−)) by single daily oral gavage for 8 weeks. Blood glucose levels in *db/db* (−) mice gradually increased and were significantly higher than those of *db/db*-HD mice (Fig. 1a); however, no significant difference was observed between *db/db*-LD mice and *db/db* (−) mice. In contrast to previous findings on the antihypertensive effects of SGLT2i^18–20^, no significant difference was noted in systolic BP between *db/db* mice treated with/without ipragliflozin (Fig. 1b). Body weights gradually increased in all *db/db* mice groups, and were significantly lower in *db/db*-HD mice than in *db/db* (−) mice (Fig. 1c). Daily urine volumes were significantly larger in *db/db* (−) mice than in ipragliflozin-treated *db/db* mice (Fig. 1d), indicating that high blood glucose and its subsequent diuretic response overcame the effects of SGLT2i. Regarding the circulating fluid volume, echocardiography at the end of the experimental period showed that ipragliflozin reduced the left ventricular chamber size from that in *db/db* (−) mice (Fig. 1e,f), suggesting that the circulating fluid volume was depleted even by the low dose of ipragliflozin.

### Ipragliflozin reduces albuminuria and renal histology in *db/db* mice

**Figure 2:**
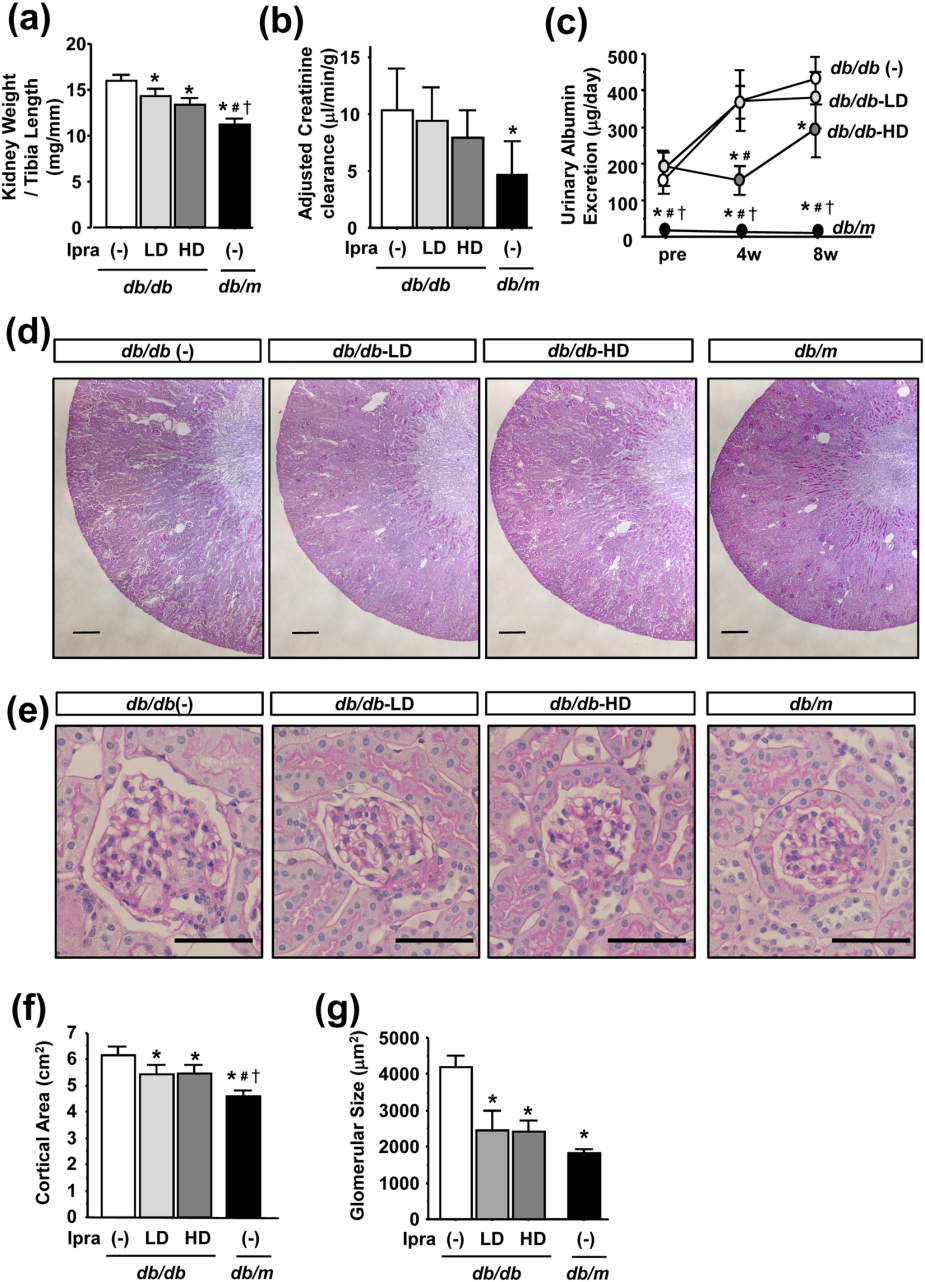
Effects of ipragliflozin on the renal phenotype of *db/db* mice. (**a**) The adjusted kidney weight of ipragliflozin-treated *db/db* mice was significantly lower than that of *db/db* (−). (**b**) Creatinine clearance adjusted by body weight slightly decreased in ipragliflozin-treated *db/db* mice. (**c**) High-dose ipragliflozin reduced urinary albumin excretion in *db/db* mice. (**d**,**e**) PAS staining of kidneys showed that the ipragliflozin treatment inhibited diabetes-induced renal hypertrophy and glomerulomegaly. (**f**,**g**) The ipragliflozin treatment reduced the cortical area and glomerular size in *db/db* mice. Data show the means ± SD, *p < 0.05 vs *db/db* (−), ^#^p < 0.05 vs *db/db*-LD, ^†^p < 0.05 vs *db/db*-HD, Bar = 500 μm in (**d**) and 50 μm in (**e**). n = 4–5 mice in each group, one-way ANOVA, Tukey’s multiple comparison.

Regarding renal outcomes, kidney weights adjusted by tibia lengths were significantly higher in *db/db* (−) mice than in *db/m* mice, and this was ameliorated by ipragliflozin in a dose-dependent manner (Fig. 2a). Creatinine clearance to evaluate glomerular hyperfiltration was significantly higher in *db/db* (−) mice than in *db/m* mice, and ipragliflozin slightly reduced creatinine clearance in a dose-dependent manner (Fig. 2b). Daily urinary albumin excretion was significantly higher in *db/db* (−) mice than in *db/db*-HD mice throughout the study period, whereas no marked difference was observed from *db/db*-LD mice (Fig. 2c).

Regarding renal histology, PAS staining of kidney sections showed that the renal cortical area was significantly enlarged in *db/db* mice, and this was ameliorated by ipragliflozin (Fig. 2d,f). Glomeruli were enlarged in *db/db* (−) mice, whereas those in ipragliflozin-treated *db/db* mice were smaller (Fig. 2e,g).

### Ipragliflozin ameliorates tubular injury in *db/db* mice

**Figure 3:**
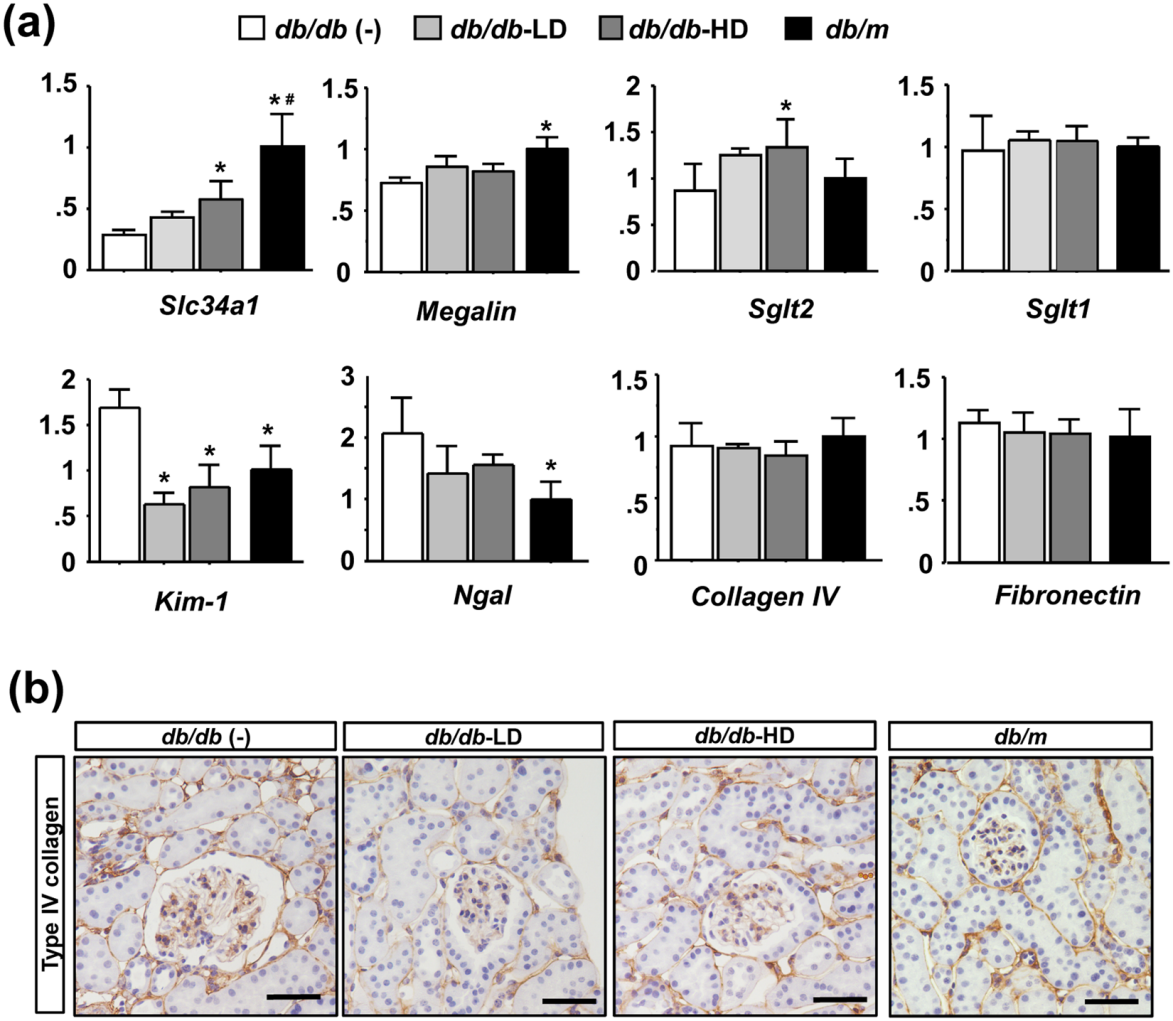
Effects of the ipragliflozin treatment on molecular changes in *db/db* mice. (**a**) qRT-PCR of SLC34a1, Megalin, SGLT1, and SGLT2 for tubular markers, KIM-1 and NGAL for tubular injury, and collagen IV and fibronectin for tissue fibrosis. (**b**) Immunostaining for type IV collagen revealed no significant differences among the study groups. Data show the means ± SD, *p < 0.05 vs *db/db* (−), Bar = 50 μm in (**b**). n = 4–5 mice in each group, one-way ANOVA, Tukey’s multiple comparison.

We investigated markers of kidney integrity, injury, and subsequent tissue fibrosis. Slc34a1, which is expressed at the brush border of mature and uninjured proximal tubular epithelia^21^, was down-regulated in *db/db* (−) mice, and this was significantly ameliorated by the ipragliflozin treatment (Fig. 3a). Megalin, which plays an essential role in albumin reabsorption^14^, was slightly down-regulated in *db/db* (−) mice, and this was slightly ameliorated by the ipragliflozin treatment. Regarding tubular injury, the expression of Kidney injury molecule-1 (KIM-1) and neutrophil gelatinase-associated lipocalin (NGAL) was up-regulated in *db/db* (−) mice, and this was inhibited by the ipragliflozin treatment (Fig. 3a). Regarding interstitial fibrosis, no significant differences were observed in type IV collagen or fibronectin expression among the experimental groups (Fig. 3a,b). Regarding inflammatory phenotypes, the mRNA amplification of IL-1beta and IL-6 was not detected by RT-PCR in any experimental group. Macrophage infiltration was evaluated by F4/80 immunostaining, which revealed that F4/80-positive macrophages were rarely present in any experimental group (Supplementary Fig. S1).

### Ipragliflozin reduces reaction oxygen species (ROS) production and oxidative stress in *db/db* mice

**Figure 4:**
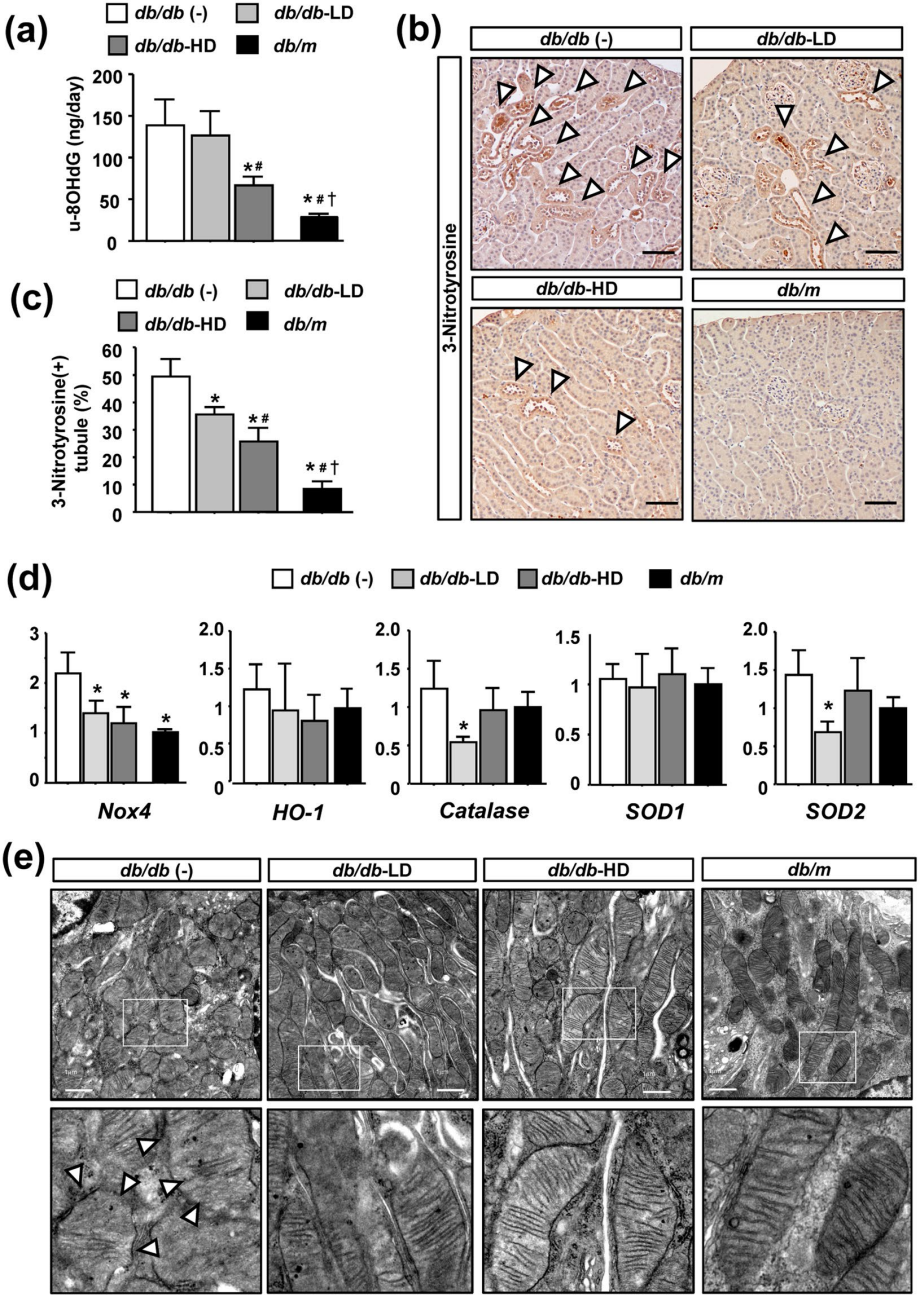
Effects of ipragliflozin on oxidative stress in *db/db* mice. (**a**) High-dose ipragliflozin reduced the urinary excretion of 8-OHdG in *db/db* mice. (**b**,**c**) Immunostaining of 3-nitrotyrosine and the quantification of positive tubules showed that the ipragliflozin treatment reduced its expression in tubular epithelia in *db/db* mice. Arrowheads indicate nitrotyrosine-positive tubules. (**d**) High- and low-dose ipragliflozin prevented the up-regulation of Nox4; however, no significant differences were observed in the mRNA expression of heme oxygenase-1 (HO-1), catalase, or superoxide dismutase-1 (SOD1) or −2 (SOD2) in the kidneys of *db/db* mice. (**e**) Transmission electron microscopy of tubular epithelia showed that the mitochondria of *db/db* mice contained peripherally-located short cristae and were replaced by a homogenized matrix, which was ameliorated by the ipragliflozin treatment. Lower panels are high magnification pictures of the small squares in the upper panels. Data show the means ± SD, *p < 0.05 vs *db/db* (−), ^#^p < 0.05 vs *db/db*-LD, ^†^p < 0.05 vs *db/db*-HD, Bar = 50 μm in (**b**) and 1 μm in (**e** upper). n = 4–5 mice in each group, one-way ANOVA, Tukey’s multiple comparison.

In order to elucidate the molecular mechanisms responsible for the renoprotective effects of ipragliflozin, we focused on ROS overproduction and consequent oxidative stress in diabetic kidneys. The urinary excretion of 8-hydroxy-2′-deoxyguanosine (8-OHdG), a major form of DNA damage induced by ROS, was significantly elevated in *db/db* (−) mice, and this was ameliorated by a high dose of ipragliflozin (Fig. 4a). Immunostaining of 3-nitrotyrosine to evaluate the localization of ROS overproduction in the kidneys revealed positive staining in proximal tubular epithelia in *db/db* (−) mice (Fig. 4b) and also that the ipragliflozin treatment reduced positive staining in a dose-dependent manner (Fig. 4c). NADPH oxidase 4 (Nox4), a major source of ROS in the kidneys^22^, was significantly up-regulated in *db/db* (−) mice, and this was ameliorated by high- and low-dose ipragliflozin (Fig. 4d). In contrast, regarding antioxidants, high-dose ipragliflozin did not exert any significant effects on the mRNA expression of heme oxygenase-1 (HO-1), catalase, or superoxide dismutase-1 (SOD1) or −2 (SOD2). Since mitochondria are also considered to be a major source of ROS production in diabetic kidneys^15^, we investigated the ultrastructure of mitochondria in tubular epithelia by transmission electron microscopy (TEM)^23^ (Fig. 4e). In the proximal tubular epithelia of *db/m* mice, regular crista formation was preserved and a homogenous inner matrix was noted. In contrast, the mitochondria of *db/db* (−) mice contained peripherally-located short cristae and were replaced by a homogenized matrix. Ipragliflozin-treated *db/db* mice showed firm and regular crista formation, indicating less mitochondrial injury than *db/db* (−) mice.

### Ipragliflozin reduces glomerular injury and oxidative stress in *db/db* mice

**Figure 5:**
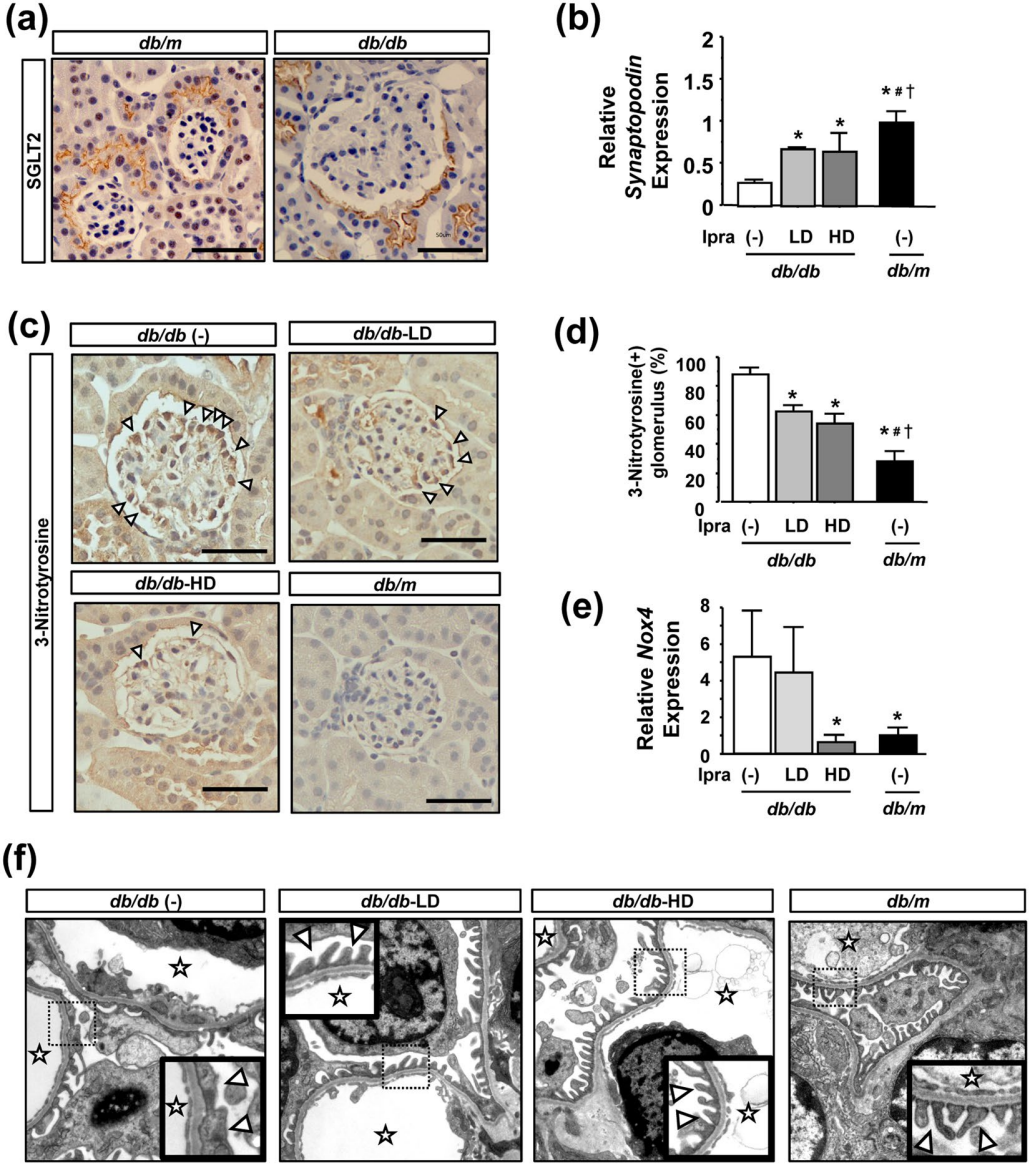
Effects of ipragliflozin on glomerular injury in *db/db* mice. (**a**) Immunostaining for SGLT2 showed negative staining within glomeruli in *db/m* and *db/db* mice. (**b**) Ipragliflozin ameliorated reductions in podocyte-specific gene expression in *db/db* mice. (**c**,**d**) Immunostaining of 3-nitrotyrosine and the quantification of positive glomeruli showed that ipragliflozin reduced its expression within glomeruli in *db/db* mice. Arrowheads indicate nitrotyrosine-positive glomerular cells. (**e**) High-dose ipragliflozin prevented the up-regulation of Nox4 in glomeruli isolated from *db/db* mice. (**f**) Transmission electron microscopy of glomerulus showed that the foot process effacement of podocytes was ameliorated by the ipragliflozin treatment. Magnified pictures of dotted squares are shown in the small squares. Stars indicate glomerular capillary lumens. Arrowheads indicate podocyte foot processes. Data show the means ± SD, *p < 0.05 vs *db/db* (−), ^#^p < 0.05 vs *db/db*-LD, ^†^p < 0.05 vs *db/db*-HD, Bar = 50 μm in (**a**) and (**c**). n = 4–5 mice in each group, one-way ANOVA, Tukey’s multiple comparison.

In order to examine the impact of the ipragliflozin treatment on glomerular injury in *db/db* mice in more detail, we isolated glomeruli by the systemic perfusion of Dynabeads solution (Supplementary Fig. S2a). We confirmed the specificity of this glomerulus isolation method by the significant up-regulation of nephrin and synaptopodin expression, markers of mature podocytes, and the significant down-regulation of SLC34a1 expression, a marker only expressed in terminally differentiated proximal tubular epithelia^21^ (Supplementary Fig. S2b). Immunostaining of SGLT2 revealed no expression within the glomeruli in *db/m* and *db/db* mice, except in glomerular parietal cells (Fig. 5a).

qPCR using RNA samples from isolated glomeruli revealed that the mRNA expression of synaptopodin was reduced in *db/db* mice, and this was ameliorated by the ipragliflozin treatment (Fig. 5b). We then investigated glomerular oxidative stress and found positive staining for nitrotyrosine mainly in podocytes located outside of glomerular capillaries in diabetic mice (Fig. 5c). The quantification of nitrotyrosine-positive glomeruli showed positive staining in approximately 80% of the glomeruli of *db/db* mice, and a dose-dependent reduction in its frequency by ipragliflozin was noted (Fig. 5d). Nox4 expression within glomeruli was significantly up-regulated in *db/db* (−) mice, and this was ameliorated by high-dose ipragliflozin (Fig. 5e). Ultrastructural analyses of glomeruli by TEM revealed that the ipragliflozin treatment preserved foot process formation in *db/db* mice; however, foot process effacement was prominent in *db/db* (−) mice (Fig. 5f).

### Short-term ipragliflozin reduces renal tissue hypoxia in db/db mice

**Figure 6:**
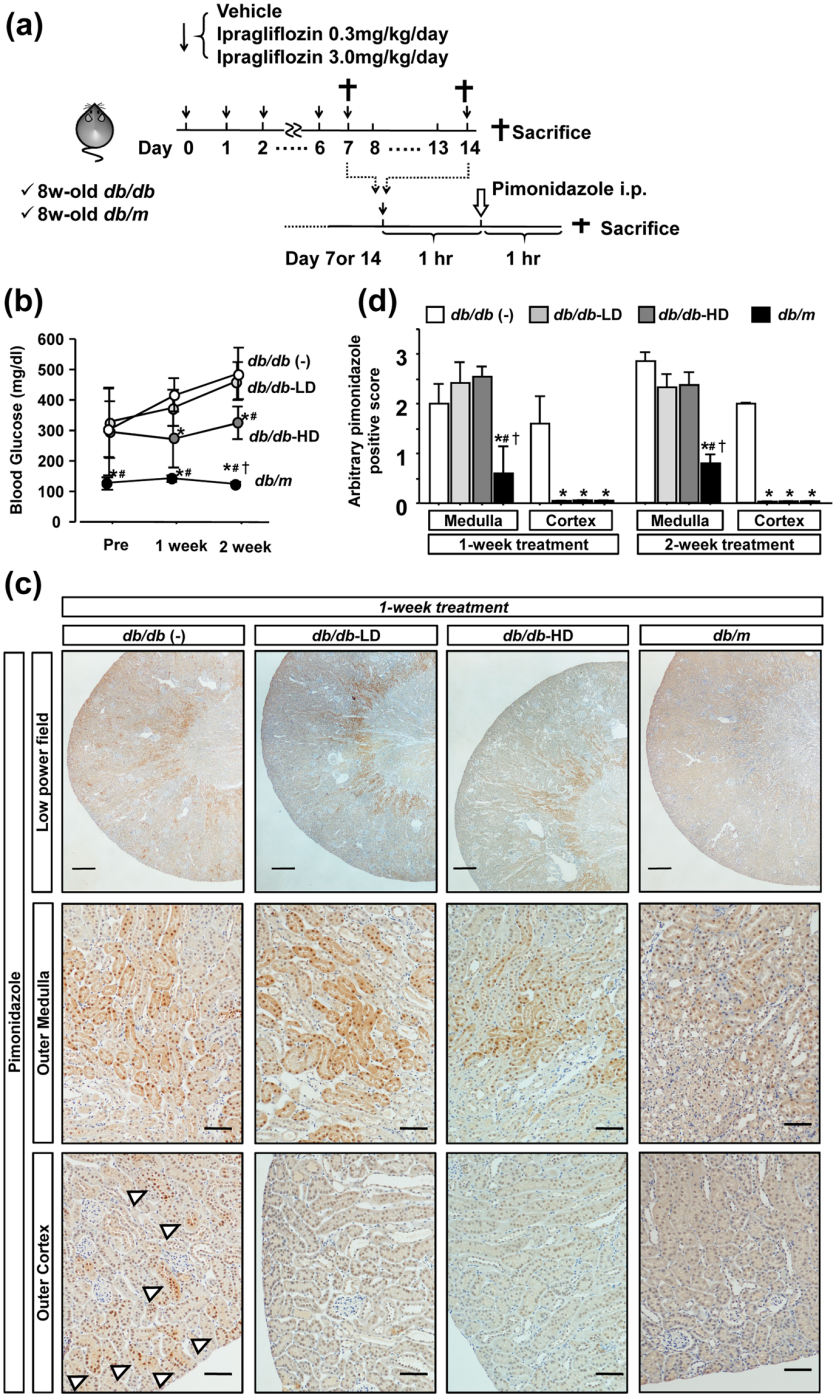
Effects of a short-term ipragliflozin treatment on renal hypoxia in *db/db* mice. (**a**) The design of the experiment. (**b**) A short-term treatment with high-dose ipragliflozin lowered blood glucose levels in *db/db* mice. (**c**) Positive staining for pimonidazole was detected in the outer medulla in db/db mice. (**d**) Both one- and two-week administration of ipragliflozin reduced positive staining for pimonidazole in the outer cortex, not in the outer medulla. Data show the means ± SD, *p < 0.05 vs *db/db* (−), ^#^p < 0.05 vs *db/db*-LD, ^†^p < 0.05 vs *db/db*-HD, Bar = 100 μm in a low power field picture and 50 μm in other pictures in (**c**). n = 4–5 mice in each group, one-way ANOVA, Tukey’s multiple comparison.

A previous study demonstrated that diuretics increased renal tissue oxygenation through their inhibitory effects on sodium reabsorption^24^. Due to its similar effects, we investigated whether ipragliflozin improves parenchymal tissue oxygenation in diabetic mouse kidneys in order to elucidate the mechanisms responsible for the amelioration of proximal tubular injury by ipragliflozin. In chronic kidney disease, parenchymal hypoxia is commonly observed regardless of its original pathogenesis, and is known as the final common pathway of kidney disease^25^. In order to minimize the effects of chronic kidney injury by hyperglycemia on kidney tissue hypoxia, we analyzed the effects of a short-term ipragliflozin treatment on renal tissue oxygenation (Fig. 6a).

After one- and two-week treatments with ipragliflozin, blood glucose levels were significantly reduced in *db/db*-HD mice, but not in *db/db*-LD mice (Fig. 6b). We performed pimonidazole immunostaining to detect tissue hypoxia. In *db/m* mice, the pimonidazole-positive area was limited to within the cortico-medullary junction at which oxygen tension is the lowest under physiological conditions due to the countercurrent exchange properties of the vasa recta^26^ (Fig. 6c,d, Supplementary Fig. S3). In *db/db* mice, the pimonidazole-positive area expanded towards the outer cortex (Fig. 6c,d, Supplementary Fig. S3). In contrast, a pimonidazole-positive area was rarely detected in the cortex of ipragliflozin-treated *db/db* mice, whereas that in the medulla was similar to *db/db* mice (Fig. 6c,d, Supplementary Fig. S3), indicating that the effects of ipragliflozin on tissue oxygenation are more prominent in the cortex in which SGLT2 expression is predominantly found.

### Ipragliflozin exerts renoprotective effects in a Streptozocin (STZ)-induced type 1 diabetes model

**Figure 7:**
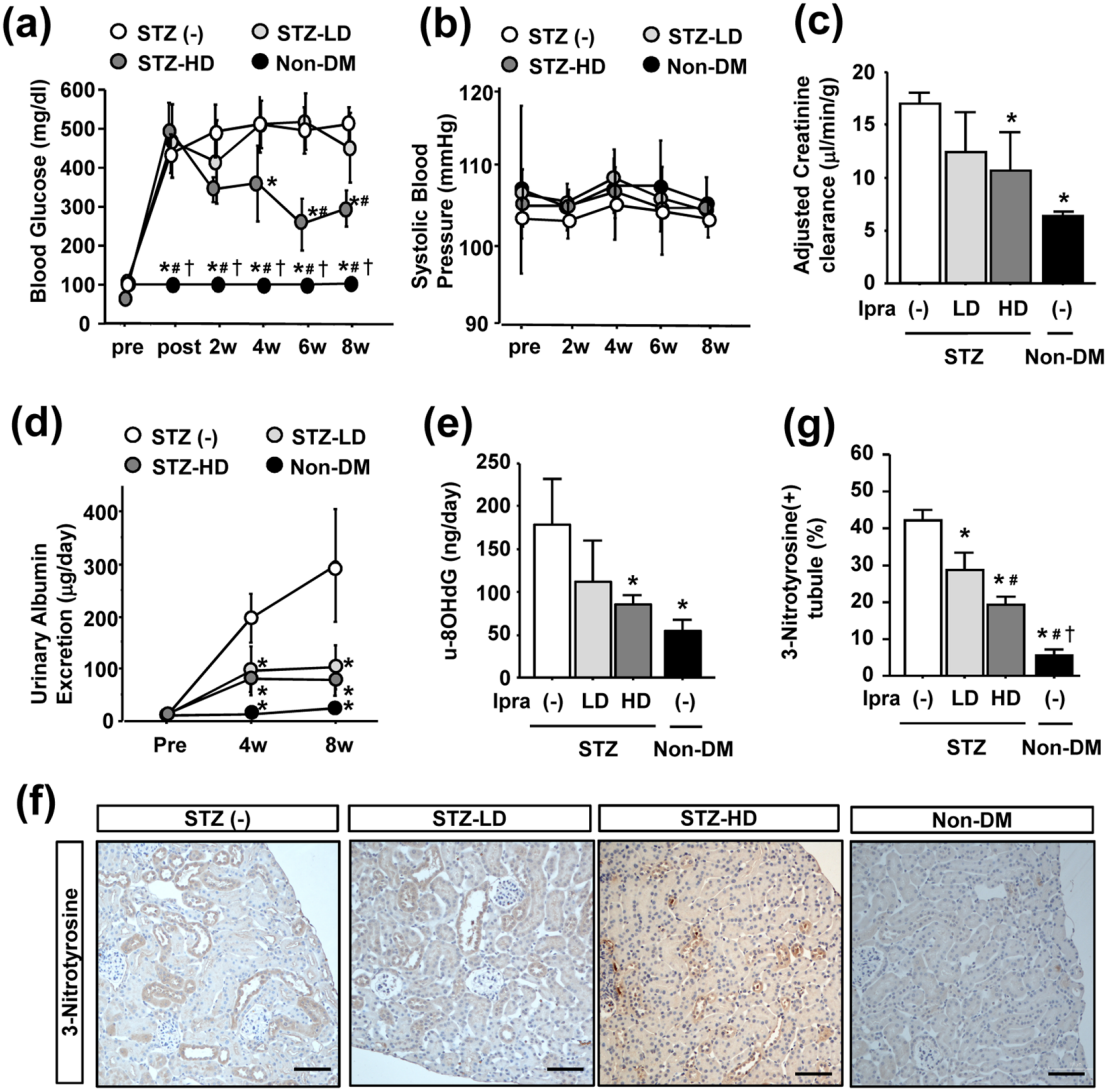
Effects of ipragliflozin on renal phenotypes in STZ-induced type 1 diabetic mice. (**a**) High-dose ipragliflozin significantly inhibited increases in blood glucose levels in STZ-injected mice. (**b**) The ipragliflozin treatment did not affect systolic blood pressure. (**c**) Ipragliflozin slightly reduced creatinine clearance adjusted by body weight decreases in STZ-injected mice in a dose-dependent manner. (**d**) High- and low-dose ipragliflozin reduced urinary albumin excretion in STZ-injected mice. (**e**) High-dose ipragliflozin reduced the urinary excretion of 8-OHdG in STZ-injected mice. (**f**,**g**) Immunostaining of 3-nitrotyrosine and the quantification of its positive tubules showed that the ipragliflozin treatment reduced its expression in tubular epithelia in STZ-injected mice. Arrowheads indicate nitrotyrosine-positive tubules. Data show the means ± SD, *p < 0.05 vs STZ (−), ^#^p < 0.05 vs STZ-LD, ^†^p < 0.05 vs STZ-HD, Bar = 100 μm in (**e**), n = 4–5 mice in each group, one-way ANOVA, Tukey’s multiple comparison.

In order to establish whether ipragliflozin exerts renoprotective effects not only in a type 2, but also in a type 1 diabetes model, we administered ipragliflozin to a STZ-induced type 1 diabetes model. One week after the STZ injection, mice with blood glucose levels that increased to higher than 350 mg/dl were used in experiments. Similar to the experiments on *db/db* mice, four mouse groups were included in this experiment: non-diabetic controls (non-DM) and STZ-injected mice treated with high- or low-dose ipragliflozin (3 mg/kg/day: STZ-HD, 0.3 mg/kg/day: STZ-LD, respectively) or vehicle (STZ (−)) by single daily oral gavage for 8 weeks. Blood glucose levels in STZ (−) mice gradually increased and were significantly higher than those in STZ-HD mice (Fig. 7a); however low-dose ipragliflozin did not reduce blood glucose levels. No significant differences were observed in systolic BP between STZ mice treated with/without ipragliflozin (Fig. 7b). Body weights gradually decreased in all STZ mice groups, with no significant difference being noted among the groups (Supplementary Fig. S4a). Daily urine volumes were significantly larger in STZ (−) mice than in ipragliflozin-treated STZ mice (Supplementary Fig. S4b).

Regarding renal outcomes, no significant difference was observed in kidney weights adjusted by tibia lengths among any experimental group (Supplementary Fig. S4c). Creatinine clearance to evaluate glomerular hyperfiltration was significantly higher in STZ (−) mice than in non-DM mice, and ipragliflozin slightly reduced creatinine clearance in a dose-dependent manner (Fig. 7c). In contrast to the experiments on *db/db* mice, daily urinary albumin excretion was significantly higher in STZ (−) mice than in other ipragliflozin-treated STZ mice throughout the study period, and no significant difference was noted between STZ-HD and STZ-LD mice (Fig. 7d).

Regarding ROS overproduction and consequent oxidative stress, the urinary excretion of 8-OHdG was significantly elevated in STZ (−) mice, and this was ameliorated by ipragliflozin in a dose-dependent manner (Fig. 7e). Immunostaining of nitrotyrosine revealed positive staining in tubular epithelia in STZ (−) mice (Fig. 7f). The quantification of nitrotyrosine-positive tubules showed that the ipragliflozin treatment reduced positive staining in a dose-dependent manner (Fig. 7g). However, in contrast to the experiments on *db/db* mice, 3-nitrotyrosine-positive cells were rarely found within glomeruli in any experimental group (Supplementary Fig. S5).

## Discussion

**Figure 8:**
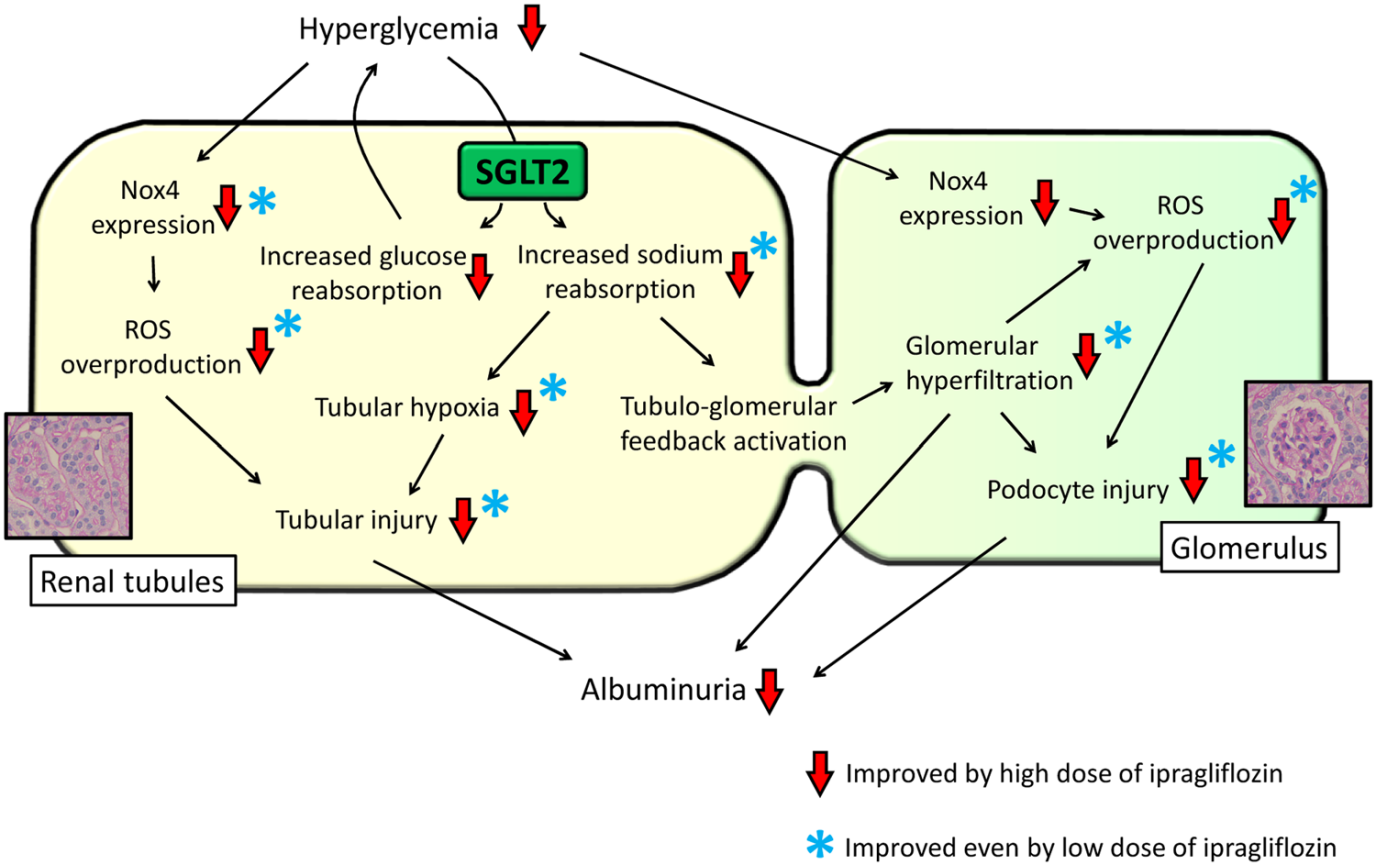
Scheme of effects of ipragliflozin on early diabetic nephropathy. Red arrows indicate the phenotypes reduced by high-dose ipragliflozin. Blue asterisks indicate the phenotypes reduced even by low-dose ipragliflozin.

The present results demonstrate that ipragliflozin exerts renoprotective effects through multifactorial pathways, which may explain the mechanisms responsible for its reported favorable clinical outcomes. In the present study, abnormal glomerular phenotypes, such as glomerulomegaly and the subsequent loss of mature podocyte markers, were reduced even by low-dose ipragliflozin. Another novel result of the present study is that ipragliflozin reduced tissue hypoxia in the kidney cortex. This result suggests that ipragliflozin exerts pleiotropic renoprotective effects by improving renal cortical hypoxia and ameliorating the abnormal hemodynamics observed in early diabetic nephropathy (Fig. 8). In contrast, dose-dependent decreases were noted in the overproduction of ROS and consequent tubular injury in *db/db* mice, indicating that the blood glucose-lowering effects of ipragliflozin are essential for renoprotective mechanisms through oxidative stress-mediated pathways.

The overproduction of ROS and subsequent increase in oxidative stress play important roles in the development of kidney disease^15^. Nox family members are major sources of ROS, and Nox4 is predominantly expressed in the kidneys^22^. Nox4 localizes within mitochondria^27^ and produces a large amount of ROS, which is reportedly larger than that produced by other Nox subtypes^22,28^. Consistent with the present results, Nox4 is up-regulated under diabetic conditions, which contributes to the overproduction of ROS and subsequent progression of diabetic kidney disease^28^. Upstream effectors of Nox4 expression in diabetic kidneys have been proposed, and these signaling pathways differ among cell types^29^. Our experiments using total kidney samples showed that Nox4 expression and subsequent ROS production were inhibited by ipragliflozin in tubular epithelia, which is consistent with a previous study^7^. Its dose-dependent inhibitory effects suggested that the blood glucose-lowering effects of ipragliflozin are crucial for suppressing the overproduction of ROS in tubular epithelia.

Regarding podocytes, Nox4 and subsequent ROS overproduction also play a critical role in their injury, and this is accompanied by the loss of podocytes or foot process effacement^30^, as observed in our experiments. You *et al*. recently reported that the podocyte-specific induction of Nox4 exhibited similar glomerular phenotypes to diabetic kidney disease, suggesting that Nox4 plays an essential role in glomerular injury^31^. In our experiments using isolated glomerular samples, low- and high-dose ipragliflozin reduced synaptopodin expression and subsequent oxidative stress within glomeruli; however, only high-dose ipragliflozin ameliorated the diabetes-induced up-regulation of Nox4 in glomeruli. These results suggest that glycemic control by high-dose ipragliflozin is needed in order to ameliorate Nox4 expression in podocytes, similar to tubular epithelia. In contrast, a previous study demonstrated that dapagliflozin improved glomerular hyperfiltration, and this was independent of its blood glucose-lowering effects^10^. Consistent with this finding, the reductions observed in glomerular size and creatinine clearance even by low-dose ipragliflozin, suggesting the amelioration of glomerular hyperfiltration, may contribute to the preservation of podocyte integrity. In addition, based on the negative expression of SGLT2 within glomeruli, the effects of ipragliflozin on glomerular cells are not attributed to its direct SGLT2 inhibitory effects, but to the reductions it induces in blood glucose levels and its amelioration of glomerular hyperfiltration.

Due to their anatomical structure and physiological function, the kidneys are one of the most hypooxygenic organs in the whole body^32^. The reabsorption of large amounts of sodium through sodium-potassium ATPase requires extensive energy production, which subsequently increases oxygen consumption. Renal oxygen tension is markedly lower in the cortex and medulla in diabetic kidneys than in non-diabetic kidneys^33,34^. This may be due, in part, to an increase in sodium reabsorption through SGLTs, which accelerates the oxygen demands of the kidneys based on previous findings showing the ameliorating effects of phlorizin, a potent SGLT inhibitor, on renal hypoxia in the cortex^17^. Brezis *et al*. reported that the administration of diuretics increased tissue oxygen, and that this effect depended on the region at which diuretics act^24^. Based on these findings, the present results suggest that the inhibition of sodium reabsorption by ipragliflozin in the cortex contributes to the amelioration of renal hypoxia in diabetic mice.

Reductions in blood pressure closely correlated with the amelioration of albuminuria in type 2 diabetes patients^35^. Several clinical trials have shown that SGLT2i exert blood pressure-lowering effects through their diuretic actions, which may partially contribute to the amelioration of albuminuria in diabetic patients^18^. However, although echocardiography in the present study showed that high-dose ipragliflozin reduced the left ventricular chamber size, a marker of circulating fluid volume, blood pressure in ipragliflozin-treated *db/db* mice was similar to that in *db/db* (−) mice. Consistent with our results, previous rodent experiments also demonstrated that SGLT2i did not lower blood pressure in diabetic mice^7,36,37^. The reason for this discrepancy between human clinical trials and rodent experimental findings is unclear from our experiments, but may be partially caused by the time at which blood pressure was measured. In previous studies, ambulatory blood pressure monitoring under the administration of SGLT2i to diabetic patients revealed that blood pressure was only reduced during the daytime and their effects were less prominent during the nighttime^19,20^. In our experiments, we measured blood pressure at 5 pm only with a 2-week interval; therefore, we may have underestimated the blood pressure-lowering effects of ipragliflozin. Blood pressure measurements at various time points may resolve this discrepancy.

We did not provide any evidence for decreases in advanced diabetic nephropathy by ipragliflozin, which is a potential limitation of our experiments. In addition, we performed experiments using *db/db* mice as a type 2 diabetic model and STZ-injected mice as a type 1 diabetic model; however, it is unclear whether the renoprotective effects of ipragliflozin may be generalized to other diabetic mouse models such as the Akita mouse. One difficulty associated with investigating diabetic nephropathy in rodents is the diversity of susceptibilities to hyperglycemia among different strains or types of diabetes^38^. In addition, because of the shorter duration of exposure to high glucose levels, diabetic rodent models rarely exhibit the severe histological phenotypes observed in humans with advanced diabetic nephropathy^38^. In the case of advanced diabetic nephropathy, since the nephrons, including proximal tubular epithelia at which SGLT2i mainly acts, are lost, it currently remains unclear whether SGLT2i really reduced these severe phenotypes.

Another potential limitation is that the mechanisms by which signaling downstream of SGLT2 affects the intracellular pathways described herein remain unclear; however, we proposed several mechanisms by which ipragliflozin ameliorates the progression of early diabetic nephropathy in rodents. In addition, although various classes of SGLT2i have been introduced and exhibit their own selectivities for SGLT2 inhibitory effects, it has not yet been clarified whether differences in its selectivity influence renoprotective effects. *In vitro* experiments using several classes of SGLT2i may resolve this issue; however, fewer *in vitro* experiments on SGLT2 have been published^12,39^ than *in vivo* and clinical investigations. A major reason for this is that, even in transfected cells, SGLT2 expression on the cell surface and its transport activity are not sufficient to analyze downstream signaling pathways^40,41^. A recent study reported that membrane-associated protein 17 is crucial for the localization of SGLT2 at the cell surface, and that the co-transfection of its expression vector is necessary for maintaining sufficient glucose uptake through SGLT2^42^. However, it has not yet been established whether this artificial condition still reflects physiological phenotypes *in vivo*.

In conclusion, we herein confirmed dose-dependent differences in the effects of ipragliflozin on early diabetic nephropathy *in vivo*. Low-dose ipragliflozin reduced renal cortical hypoxia and the abnormal hemodynamics observed in early diabetic nephropathy. In addition to these effects, high-dose ipragliflozin exhibited renoprotective effects through a reduction in oxidative stress in tubular epithelia and glomerular podocytes. These comprehensive renoprotective effects of ipragliflozin may explain the mechanisms responsible for the prevention of renal events, as indicated by a recent large scale clinical trial^6^. However, it has not yet been elucidated whether ipragliflozin reduces the phenotypes of advanced diabetic nephropathy and, thus, further studies are warranted.

## Methods

### Animal experiments

We purchased male diabetic BKS.Cg-Dock7^m^ + / + Lepr^db^/J (*db/db*) mice and non-diabetic heterozygote (*db/m*) mice aged 8 weeks from Oriental Bio Service Inc. (Kyoto, Japan). Mice were housed under a 12-h light/dark cycle with free access to tap water and standard chow (CE-2, CLEA Japan, Inc., Tokyo, Japan).

Regarding the type 1 diabetes model, we injected 200 mg/kg of STZ intraperitoneally into 8-week-old male BALB/c mice. One week after the injection, mice with blood glucose levels higher than  350 mg/dl were analyzed.

Different doses (0.3 or 3.0 mg/kg/day) of ipragliflozin, kindly provided by Astellas Pharma Inc. (Tokyo, Japan), were administered to *db/db* mice by daily oral gavage using 0.5% methyl cellulose as a vehicle at 17:00 starting at the age of 9 to 10 weeks. Similarly, vehicle was administered to control *db/db* mice and control *db/m* mice for the same period. After 8 weeks for long-term experiments, or 1 to 2 weeks for short-term experiments after drug administration, mice were euthanized and kidney weights and tibia lengths were measured. Each experimental group contained 5 mice. All experiments were approved by the Experimental Animals Committee, Kyoto Prefectural University of Medicine, and were performed in accordance with the institutional guidelines and Guidelines for Proper Conduct of Animal Experiments by the Science Council of Japan.

### Metabolic data

Body weights, blood glucose levels, and BP were measured at 17:00 every 2 weeks. Blood glucose levels and BP were measured by a glucometer (Glutest Every, Sanwa Kagaku Kenkyusho Co., Ltd., Aichi, Japan) and non-invasive tail cuff method (BP-98A, Softron Co., Ltd., Tokyo, Japan), respectively.

Regarding 24-hour urine collection, mice were placed individually into metabolic cages (KN-645, Natsume Seisakusho Co., Ltd., Tokyo, Japan) every 4 weeks. Urine albumin, creatinine, and 8-OHdG levels were measured using an immunoturbidimetric method (Oriental Yeast Co., Ltd., Tokyo, Japan) and enzyme-linked immunosorbent assay (Nikken Seil Co., Ltd., Shizuoka, Japan), respectively.

### Tissue preparation and histological analysis

The kidneys were fixed with 4.0% paraformaldehyde and embedded in paraffin. Paraffin-embedded tissues were cut into 4-μm-thick sections. Morphological evaluations of the kidneys were performed by periodic acid-Schiff staining under standard conditions. The cortical area of the short axis section, which was cut at the middle of the kidney, was measured. The glomerular area in the same section, which contains approximately 100 glomeruli, was measured. Histological quantification was performed using a BZ-X700/BZ-X710 microscope (Keyence Corporation, Osaka, Japan) and the mean area was calculated as previously described^43^.

### Immunohistochemistry

After deparaffinization and antigen retrieval, endogenous peroxidase was quenched with 3.0% hydrogen peroxide in methanol for 20 minutes. Blocking was performed using 3.0% BSA in PBS for 30 minutes. Regarding nitrotyrosine staining, blocking was performed using 1.0% fish gelatin in PBS instead of 3.0% BSA in PBS. Sections were sequentially incubated with primary antibodies as shown in Supplementary Table S1, followed by an incubation with HRP-conjugated secondary antibodies (Supplementary Table S1). Sections were labeled with diaminobenzidine chromogenic substrate (K3468, Agilent Technologies, Inc., Santa Clara, CA), which was used for color visualization, followed by counterstaining with hematoxylin. All sections were observed using an Eclipse E600 microscope (Nikon Corporation, Tokyo, Japan).

In order to quantify oxidative stress-induced damage, nitrotyrosine-immunostained sections were analyzed. The proportions of nitrotyrosine-positive glomeruli and tubules were calculated by dividing the number of nitrotyrosine-positive glomeruli by the total number of glomeruli and by dividing the number of nitrotyrosine-positive renal tubules by the total number of renal tubules in three non-overlapping fields of the renal cortex in each section.

### Assessment of renal tissue hypoxia

Ipragliflozin was administered to *db/db* mice by daily oral gavage for one week starting at the age of 9 to 10 weeks. Two hours before sacrifice, ipragliflozin or vehicle was administered to mice by oral gavage. One hour later, pimonidazole (Hypoxyprobe^TM^-1, Natural Pharmacia International, Inc., Burlington, MA) in saline was injected intraperitoneally at a dose of 60 mg/kg. Mice were sacrificed one hour after the administration of pimonidazole. Pimonidazole was detected by immunohistochemistry (Supplementary Table S1).

Renal hypoxia was evaluated by the immunostaining of pimonidazole as follows: the proportion of pimonidazole-positive renal tubules in the cortex was calculated by dividing the number of pimonidazole-positive renal tubules by the total number of renal tubules in each field. Hypoxia in the renal cortex was graded by scoring the percentage of pimonidazole-positive renal tubules as follows: 0, 0–9%; 1, 10–29%; 2, 30–49%; and 3, 50–100%. Hypoxia in the renal outer medulla was scored according to the following intensities: 0, negative; 1, weak; 2, moderate; and 3, strong staining, as previously described with minor modifications^44^.

### Transmission electron microscopy (TEM)

An electron microscopic examination was performed by PCL Japan, Inc. (Tokyo, Japan) as follows. Small fragments of the kidneys were fixed with 2.5% glutaraldehyde overnight and post-fixed with 2.0% osmium tetroxide for 2 hours. The kidneys were dehydrated with graded ethanol solutions and embedded in epoxy resin. Semi-thin sections were stained with methylene blue. Tissues were cut into 70- to 80-nm-thick sections, picked up on a grid mesh, and stained with uranyl acetate and lead citrate. Ultra-thin sections were analyzed mainly in the glomerular loops and proximal tubules under TEM (HT7700, Hitachi High-Technologies Corporation, Tokyo, Japan).

### Isolation of glomeruli

Glomeruli were isolated as previously described with minor modifications^45^. The surface of Dynabeads M-450 Tosylactivated beads (Life Technologies, Inc., Carlsbad, CA) was inactivated by washing with BSA and diluted in 40 mL of PBS.

Mice were anesthetized and gently perfused with 8.0 × 10^7^ Dynabeads in PBS through the left ventricle. The removed kidneys were minced and digested in 1 mg/mL collagenase A (Roche Diagnostics GmbH, Mannheim, Germany) and 1 U/mL deoxyribonuclease I (Invitrogen Corporation, Carlsbad, CA) in Hank’s balanced salt solution; HBSS (Invitrogen AB, Lidingo, Sweden) at 37 °C for one hour with agitation. The digested tissue was passed through a 100-μm cell strainer and the cell strainer was washed with 5 mL of HBSS twice. The cell suspension was centrifuged and the cell pellet was resuspended with HBSS. Glomeruli containing Dynabeads were gathered by a magnetic particle concentrator and washed twice with PBS.

### RNA extraction and qRT-PCR

Total RNA was extracted from the kidneys and isolated glomeruli using TRIzol (Life Technologies, Inc., Carlsbad, CA) and Direct-zol^TM^ RNA MiniPrep (Zymo Research Corporation., Irvine, CA). Two hundred nanograms of total RNA was reverse transcribed to synthesize cDNA using a PrimeScript RT reagent Kit with a gDNA Eraser (Takara Bio Inc., Shiga, Japan). The real-time detection of PCR products was performed using KAPA SYBR FAST qPCR Master Mix (2×) Universal (Kapa Biosystems, Wilmington, MA) and a Thermal Cycler Dice Real Time System (Takara Bio Inc., Shiga, Japan). All reactions were performed in duplicate. The primers for targets are listed in Supplementary Table S2.

### Echocardiography

Echocardiography was performed at the start and end of the study using a Vevo 2100 Imaging System (VisualSonics Inc., Toronto, Canada). During echocardiography, the heart rate of mice was maintained between 400/min and 500/min by adjusting the depth of anesthesia with isoflurane (3.0–5.0% for induction; 1.0–2.0% for maintenance). Left ventricular chamber sizes were measured from M-mode recordings.

### Statistical analysis

Results are expressed as the mean ± SD. Statistical analyses were performed using an analysis of variance and Tukey’s *post hoc* tests or unpaired *t*-tests. P values of <0.05 were considered to be significant.

## Electronic supplementary material

Supplementary Information
