# Supplementary material for: Comprehensive renoprotective effects of ipragliflozin on early diabetic nephropathy in mice
Source: Sci Rep. 2018 Mar 5;8:4029. doi: 10.1038/s41598-018-22229-5 (PMC5838225; doi:10.1038/s41598-018-22229-5)
Supplement: Supplementary file 1 — Supplementary Information [file 41598_2018_22229_MOESM1_ESM.docx]

Supplementary Information

Comprehensive renoprotective effects of ipragliflozin

on early diabetic nephropathy in mice

Michitsugu Kamezaki, Tetsuro Kusaba, Kazumi Komaki, Yohei Fushimura, Noriko Watanabe, Kisho Ikeda, Takashi Kitani, Noriyuki Yamashita, Masahiro Uehara, Yuhei Kirita, Yayoi Shiotsu, Ryosuke Sakai, Takuya Fukuda, Masahiro Yamazaki, Michiaki Fukui, Satoaki Matoba and Keiichi Tamagaki

Supplementary Figure S1. Immunostaining of F4/80.

Immunostaining of F4/80 revealed that macrophage infiltration was rarely found in all experimental groups, Bar = 50 μm.


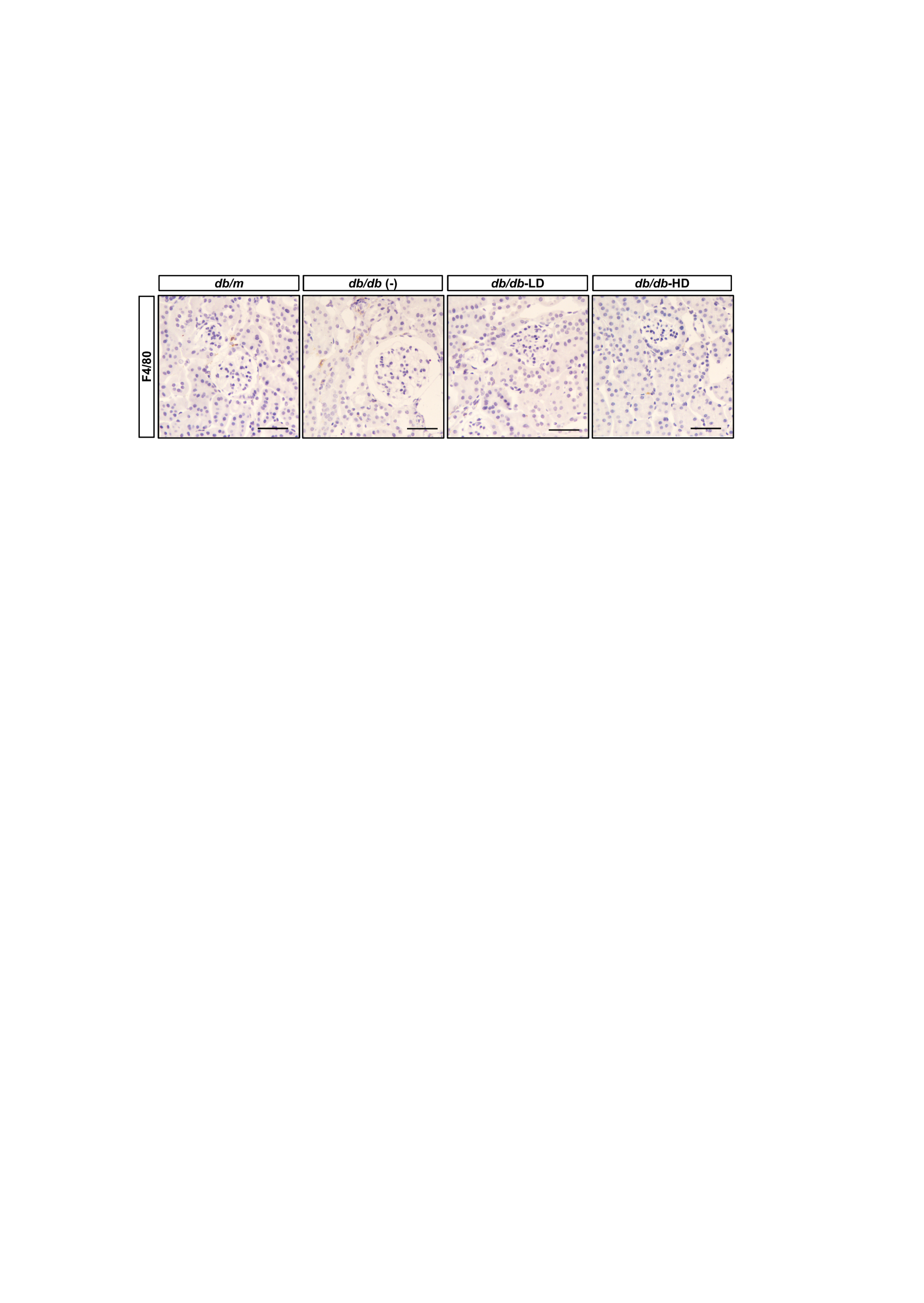


Supplementary Figure S2. Isolation of glomeruli

(a) Hematoxylin staining showed the isolated glomerulus contained the stuck dynabeads (arrowheads). (b) qPCR using RNA samples from total kidneys and isolated glomeruli showed a significant increase in podocyte-specific genes and a significant reduction of specific genes for proximal tubular epithelia. * p < 0.05, unpaired t-test, n = 5. Bar = 50 μm in (A),


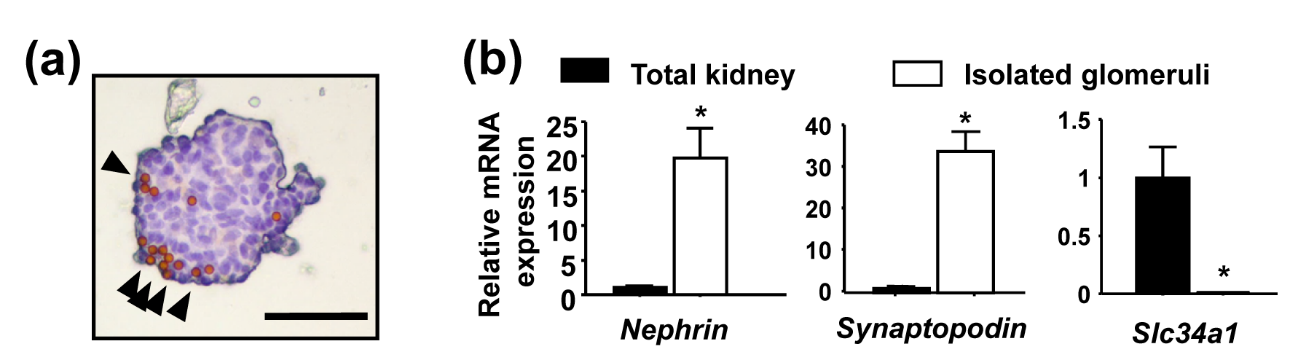


Supplementary Figure S3. Effects of ipragliflozin treatment for two weeks on renal hypoxia in *db/db* mice. Positive staining for pimonidazole was found in the outer medulla of all *db/db* mice groups. Ipragliflozin reduced positive staining for pimonidazole in the outer cortex of *db/db* mice (arrowheads). Bar = 100 μm in low power field picture and 50 μm in other pictures.


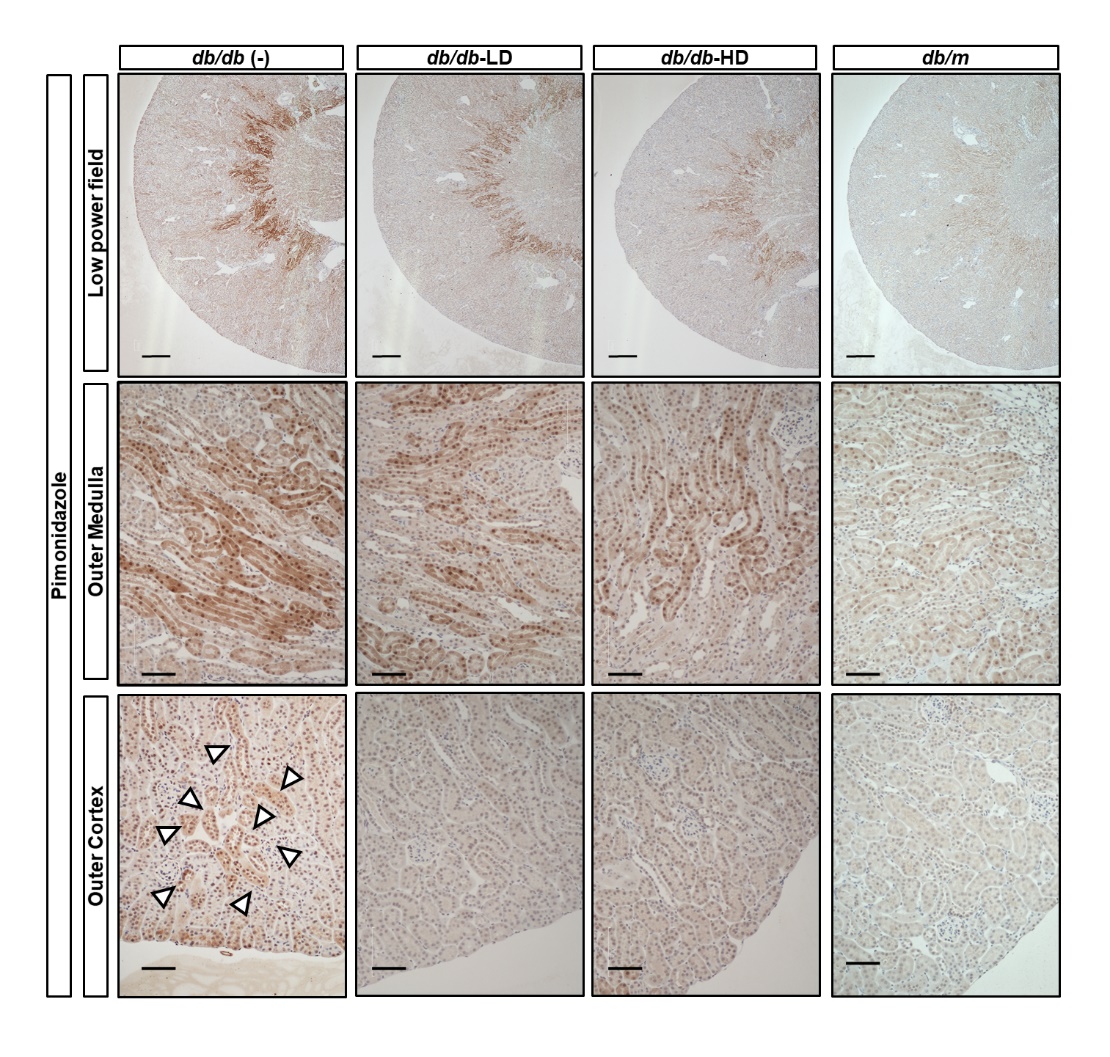


Supplementary Figure S4. Effects of ipragliflozin on the renal phenotype of STZ-injected type 1 diabetic mice. (a) Ipragliflozin treatment did not affect body weight. (b) Urine volume tended to be lower in ipragliflozin-treated STZ mice. (c)There was no difference in adjusted kidney weight among STZ mice with or without ipragliflozin treatment. Data show the means ± SD, * p < 0.05 vs STZ (-), # p < 0.05 vs STZ-LD, † p < 0.05 vs STZ-HD, n = 4-5 mice in each group, one-way ANOVA, Tukey’s multiple comparison.


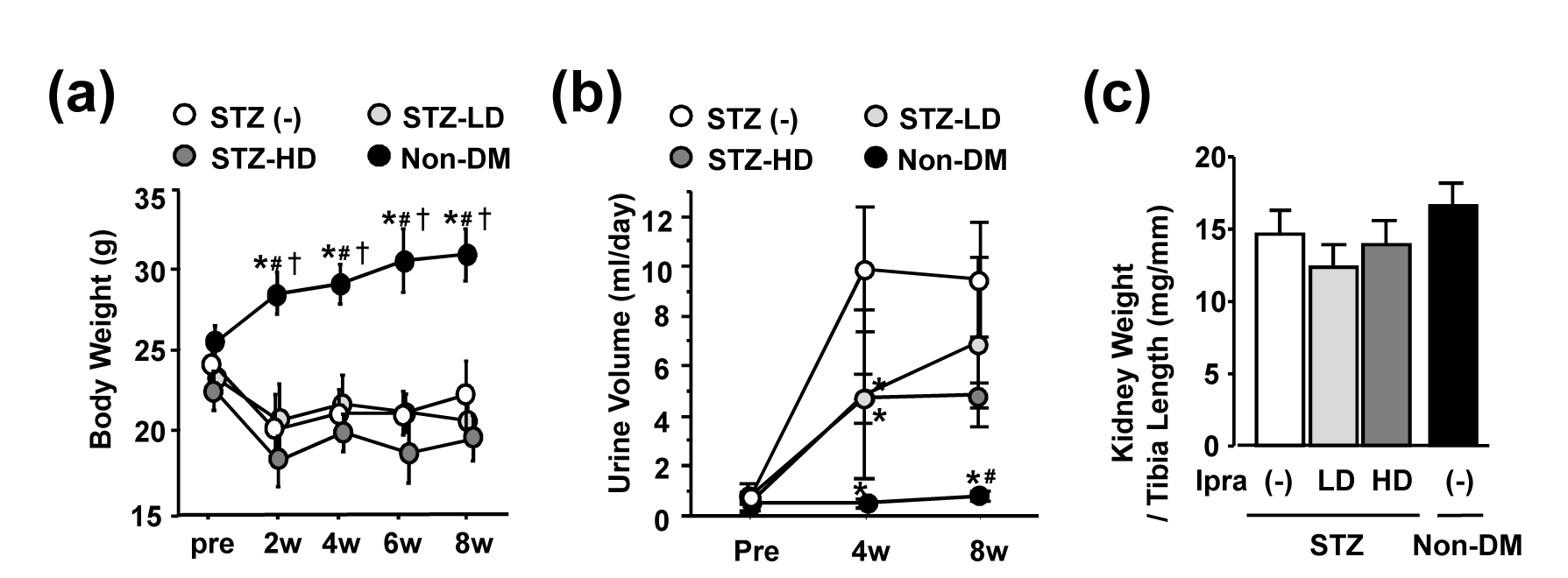


Supplementary Figure S5 Immunostaining of 3-Nitrotyrosine within glomeruli of STZ-injected type 1 diabetic mice.

There were no nitrotyrosine-positive cells within the glomeruli of STZ-injected mice. Bar = 50 μm.


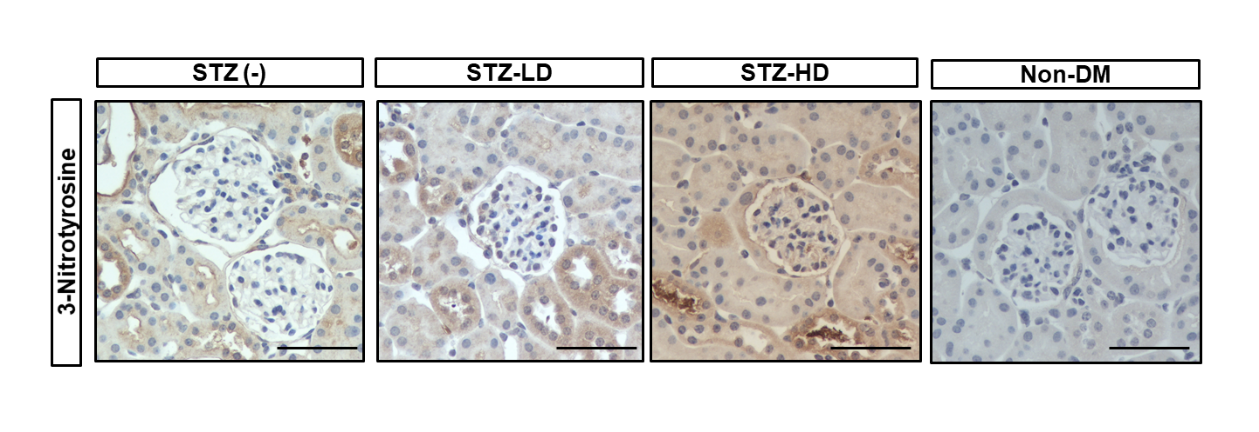


Supplementary Table S1.

List of primary and secondary antibodies for immunohistochemistry

| Antibody | Company | Number | Host | Dilution ratio |
| --- | --- | --- | --- | --- |
| Primary antibody |  |  |  |  |
| Anti-SGLT2 antibody | Sigma-Aldrich | HPA041603 | rabbit | 1:200 |
| Anti-Nitrotyrosine antibody | Merck Millipore | 06-284 | rabbit | 1:200 |
| Anti-F4/80 antibody | BioLegend | 123101 | rat | 1:200 |
| Anti-Type 4 collagen antibody | Abcam | ab6586 | rabbit | 1:600 |
| Anti-pimonidazole antibody | Natural Pharmacia International | PAb2627 | rabbit | 1:500 |
| HRP conjugated secondary antibody |  |  |  |  |
| anti-rabbit antibody | Abcam | ab80437 | goat | Not diluted |
| anti-rat antibody | Abcam | ab7097 | goat | 1:500 |

Supplementary Table S2.

List of primers for PCR

| Gene | Forward | Reverse |
| --- | --- | --- |
| *Sglt1* | ATGCGGCTGACATCTCAGTC | ACCAAGGCGTTCCATTCAAAG |
| *Sglt2* | ATGGAGCAACACGTAGAGGC | ATGACCAGCAGGAAATAGGCA |
| *Slc34a1* | ACAAAACCCTACTGGGTGGA | CTCGCTGTAGGACATCAT |
| *Megalin* | AAAATGGAAACGGGGTGACTT | GGCTGCATACATTGGGTTTTCA |
| *Kim-1* | AAACCAGAGATTCCCACACG | GTCGTGGGTCTTCCTGTAGC |
| *Ngal* | GCAGGTGGTACGTTGTGGG | CTCTTGTAGCTCATAGATGGTGC |
| *Nephrin* | GATGCGGAGTACGAGTGCC | GGGGAACTAGGACGGAGAGG |
| *Synaptopodin* | CCTGCCCGTAACTTCCGTG | GAGCGGCGGTAGGGAAAAG |
| *Col4a1* | CCTGGCACAAAAGGGACGA | ACGTGGCCGAGAATTTCACC |
| *Fibronectin* | GCTCAGCAAATCGTGCAGC | CTAGGTAGGTCCGTTCCCACT |
| *Il-1beta* | TAACCTGCTGGTGTGTGACGTT | GACAGCACGAGGCTTTTTTGT |
| *Il-6* | GACAAAGCCAGAGTCCTTCAGAGAG | CCACAAACTGATATGCTTAGGCATAAC |
| *β-actin* | AGCCATGTACGTAGCCATCC | CTCTCAGCTGTGGTGGTGAA |
| *SOD1* | ACCCGCTTCCATAAGGCTTTA | CAGCCTTGTGCCGAAAGAC |
| *SOD2* | GCCCCCTGAGTTGTTGAATA | AGACAGGCAAGGCTCTACCA |
| *Catalase* | ACATGGTCTGGGACTTCTGG | CAAGTTTTTGATGCCCTGGT |
| *HO-1* | TAAGACCGCCTTCCTGCTCAACAT | TGCTGGTTTCAAAGTTCAGGCCAC |
